# Metastatic breast cancer patients with lung or liver metastases should be distinguished before being treated with fulvestrant

**DOI:** 10.1002/cam4.2453

**Published:** 2019-08-02

**Authors:** Min He, Jun‐Jie Li, Wen‐Jia Zuo, Lei Ji, Yi‐Zhou Jiang, Xi‐Chun Hu, Zhong‐Hua Wang, Zhi‐Ming Shao

**Affiliations:** ^1^ Key Laboratory of Breast Cancer, Department of Breast Surgery Fudan University Shanghai Cancer Center, Shanghai Medical College Fudan University Shanghai China; ^2^ Department of Oncology Fudan University Shanghai Cancer Center, Shanghai Medical College Fudan University Shanghai China

**Keywords:** hormone therapy, metastatic breast cancer, prognosis, visceral metastases

## Abstract

**Background:**

Endocrine therapy is the preferred treatment for patients with hormone receptor ‐positive metastatic breast cancer (MBC). While visceral metastasis is a negative prognostic factor, few studies have distinguished between the prognoses of patients with metastases at different visceral sites.

**Patients and methods:**

In total, 398 patients receiving fulvestrant 500 mg at a single center over a 6‐year period were analyzed. Logistic regression models were used to identify the prognostic factors associated with progression‐free survival (PFS). Kaplan‐Meier analysis was used to compare the PFS of patients with lung and liver metastases.

**Results:**

Baseline visceral metastases were present in 233 patients, including 138 with lung^w/o liver^ metastases (lung metastases without liver involvement), 51 with liver^w/o lung^ metastases (liver metastases without lung involvement) and 41 with lung and liver metastases. The median PFS was 6.8 months (5.6 and 9.2 months for visceral and nonvisceral metastases, respectively, *P* = .028). PFS was longer in patients with lung^w/o liver^ metastases than in those with liver^w/o lung^ metastases or lung and liver metastases (9.6, 3.7 and 3.2 months, respectively, *P* < .001; liver^w/o lung^ vs. lung^w/o liver^ hazard ratio (HR) 1.70; lung and liver vs. lung^w/o liver^ HR 2.85). Patients with liver metastases experienced significantly worse PFS than those without liver involvement (3.7 vs. 9.2 months, *P* < .001). PFS benefits were observed in patients with longer disease‐free intervals, no liver metastases, and no previous chemotherapy.

**Conclusion:**

Fulvestrant treatment benefited patients with lung^w/o liver^ or nonvisceral metastases. When treating hormone receptor‐positive/HER2‐negative MBC patients with endocrine therapy, it is important to differentiate patients with lung metastases from those with liver metastases.

## Graphical abstract

Our results demonstrated that patients with liver metastases respond poorly to fulvestrant, while in contrast, the efficacy of fulvestrant treatment in patients with lung metastases but no liver involvement was comparative to that of patients with non‐visceral metastases. Therefore, we suggest that categorizing MBC into “liver” and “non‐liver” metastases may better reflect patient prognosis compared to “visceral” and “non‐visceral” metastasis.

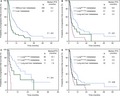

## 1 INTRODUCTION

More than 70% of patients with metastatic breast cancer (MBC) present with hormone receptor‐positive disease.^1^ Endocrine therapy is the recommended initial treatment for hormone receptor‐positive metastatic disease in the current guidelines, even with visceral metastases.^2^ Prospective trials have shown that endocrine therapy, such as fulvestrant with or without a CDK 4/6 inhibitor, may substantially improve progression‐free survival (PFS) with good patient tolerance.^3^, ^4^, ^5^, ^6^

Fulvestrant, a 17β‐estradiol analog, is an antiestrogen that suppresses estrogen signaling by binding to the estrogen receptor (ER) and inducing its degradation; it has antagonistic activity against estrogen but no agonistic effects on estrogen.^7^ Fulvestrant 500 mg was approved as the standard dose for hormone receptor‐positive MBC patients after the CONFIRM study, and the subsequent China‐CONFIRM study found that fulvestrant 500 mg had greater efficacy than fulvestrant 250 mg in patients who experienced disease recurrence or progression after previous endocrine therapy.^8^, ^9^ The FIRST and FALCON trials further demonstrated the superior efficacy of fulvestrant 500 mg compared to anastrozole in the first‐line setting for hormone receptor‐positive MBC patients.^3^, ^10^

The above trials showed that fulvestrant 500 mg was associated with improved efficacy as both a first‐ and later‐line treatment. However, PFS ranged from a few months with a very aggressive course of the disease to several years without major limitations on the quality of life. These findings underscore the importance of defining biological or clinical prognostic factors to develop individualized treatment strategies.

Patients with visceral metastases are often considered less likely to respond to hormonal therapy than those with nonvisceral metastases. In the FALCON study, the median PFS in patients with and without visceral disease was 13.8 months and 22.3 months, respectively, after first‐line treatment with fulvestrant.^2^, ^3^ Heterogeneity exists among visceral metastases, and to the best of our knowledge, few studies have evaluated differences in the efficacy of endocrine treatment according to the site of visceral metastasis.^11^ The lungs are one of the most common visceral metastatic sites; however, it is still unknown whether the sensitivity of patients with lung metastases to endocrine therapy differs from that of those with other visceral metastatic sites. The current article describes a retrospective analysis of patients treated with fulvestrant 500 mg to determine whether patients with visceral metastases (i.e., metastases to the liver and/or lungs) respond differently to treatment and to identify prognostic factors associated with improved PFS with fulvestrant treatment.

## 2 PATIENTS AND METHODS

### 2.1 Study design and patients

In this retrospective study, 505 hormone receptor‐positive MBC patients treated with fulvestrant between July 2011 and October 2017 at the Fudan University Shanghai Cancer Center (FUSCC) were enrolled. This study was approved by an independent ethics committee at the FUSCC. All patients provided written informed consent. Clinical data were retrospectively collected from the electronic medical records system.

Eligible patients were women with histologically confirmed ER‐positive and/or progesterone receptor (PgR)‐positive advanced/recurrent breast cancer. Key exclusion criteria were the presence of human epidermal growth factor receptor 2 (HER2)‐positive breast cancer (defined as an immunohistochemical staining score of 3+ or a positive fluorescence in situ hybridization result), treatment with fulvestrant 250 mg, and treatment with fulvestrant combined with other drugs, such as capecitabine, everolimus or an aromatase inhibitor (AI).

The initial site of relapse was established at baseline. Visceral metastasis was defined by baseline disease at any of the following sites: lungs, liver, pleura, brain, ovaries, pleural effusion, adrenal glands, peritoneum, esophagus or pancreas. Patients with lung or liver metastases were further categorized into three subgroups based on their sites of metastasis: lung^w/o liver^ metastases (lung metastasis without liver involvement); liver^w/o lung^ metastases (liver metastases without lung involvement); and both lung and liver metastases.

### 2.2 Treatment

Fulvestrant was administered intramuscularly in a loading‐dose regimen: 500 mg on days 0, 14, and 28 and once every 28 days thereafter.

### 2.3 Tolerability and safety

Adverse events (AEs) were determined retrospectively based on medical records. Generally, fulvestrant was well tolerated in all patients. The most frequently reported AEs were hot flashes, nausea, pain, headaches, vasodilatation, pharyngitis, bone pain, and vomiting. None of these events were sufficiently serious to terminate treatment.

### 2.4 Statistical analysis

The primary endpoint of the study was PFS, defined as the time from the start of fulvestrant treatment to objective disease progression (progression of existing disease, appearance of new lesions of disease, or death from any cause). Subjects who had not progressed at the time of the analysis were censored using the last assessment date.

A subgroup analysis of the PFS data was conducted with the following covariates: menopausal status, disease‐free interval (DFI), PgR status, bone‐only metastasis, endocrine treatment‐naïve disease, metastatic sites, prior endocrine therapy for MBC, prior chemotherapy for metastatic disease, and level of responsiveness to endocrine therapy before fulvestrant treatment (primary resistance vs. secondary resistance). Primary resistance to endocrine therapy was defined as recurrence occurring during the first 2 years of adjuvant endocrine therapy or disease progression within the first 6 months of first‐line endocrine therapy for advanced disease. Secondary resistance to endocrine therapy was defined as recurrence occurring after the first 2 years of adjuvant endocrine therapy or disease progression after the first 6 months of endocrine therapy for advanced disease.

Kaplan‐Meier plots were produced for the survival endpoints, and the median PFS was calculated. Univariate logistic regression models followed by multivariable logistic regression models were used to identify factors contributing to prolonged PFS with treatment with fulvestrant 500 mg. Hazard ratios (HRs) were obtained using the Cox proportional hazards regression models, with HR > 1 reflecting a shortened PFS. Two‐sided *P* values <.05 were considered statistically significant. All statistical analyses were carried out using SPSS version 21.0 (IBM SPSS Statistics).

## 3 RESULTS

### 3.1 Patient and disease characteristics

**Figure 1:**
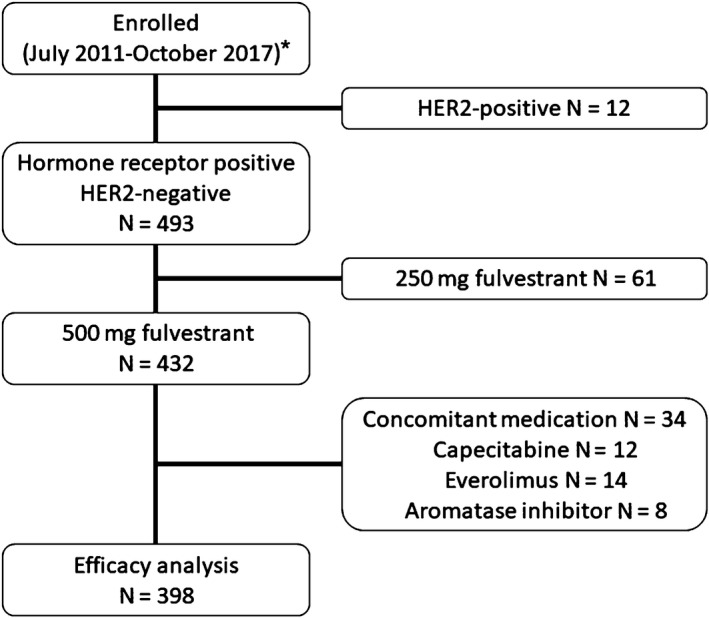
CONSORT diagram. *Fulvestrant was approved by China Food and Drug Administration in March 2011. HER2, human epidermal growth factor receptor 2

In total, 505 patients were enrolled. After excluding 107 ineligible patients, 398 patients were analyzed (Figure 1). The patients’ baseline and disease characteristics are summarized in Table 1. The median age of the patients at the start of fulvestrant treatment was 58 (range, 30‐86) years. The most common site of metastasis was bone (62.1%). Baseline visceral metastases were present in 233 patients (58.5%), and lung metastases were observed more commonly than liver metastases (179 and 92 patients, respectively). We observed lung^w/o liver^ metastases in 138 patients, liver^w/o lung^ metastases in 51 patients and both lung and liver metastases in 41 patients. The three remaining patients presented with brain metastases accompanied by soft tissue metastases and had undergone local brain radiotherapy; there was no clinical evidence of disease progression at the time of enrollment. The majority of patients with pleural metastases also presented with lung metastases (21 out of 31).

**Table 1:** Baseline patient demographics and disease characteristics

Characteristics	n = 398	%
Median age, years	58 (30‐86)	
Menopausal status		
Premenopause^a^	84	21.1
Postmenopause	314	78.9
Disease‐free interval^b^		
>5 years	181	43.0
≤5 years	171	45.5
ER status		
Positive	393	98.7
Negative	5	1.3
PgR status		
Positive	285	71.6
Negative	95	23.9
Unknown	18	4.5
Metastatic sites		
Nonvisceral	165	41.5
Bone	247	62.1
Bone only	61	15.3
Visceral disease	233	58.5
Any lung	179	45.0
Any liver	92	23.1
Lung^w/o liver^	138	34.7
Liver^w/o lung^	51	12.8
Lung + liver	41	10.3
Pleural	31	7.8
Brain^c^	12	3.0
Ovary	4	1.0
Other^d^	9	2.3
No. of disease sites		
1	122	30.6
≥2	276	69.4
De novo metastatic disease	46	11.6
Adjuvant ET		
Antiestrogen ± LH‐RH analog	172	43.2
Aromatase inhibitor ± LH‐RH analog	102	25.6
Antiestrogen followed by aromatase inhibitor	13	3.3
None	40	10.0
Unknown	24	6.0
Prior ET for metastatic disease		
No	145	36.4
Yes	253	63.6
Prior ET type for metastatic disease		
Antiestrogen ± LH‐RH analog	45	11.3
Aromatase inhibitor ± LH‐RH analog	238	59.8
Everolimus	7	1.8
Prior sensitivity to ET		
Primary resistance	71	17.8
Secondary resistance	295	74.1
Prior chemotherapy for metastatic disease		
No	203	51.0
Yes	195	49.0
Treatment immediately preceding fulvestrant		
None	32	8.0
Chemotherapy	102	25.6
Antiestrogen ± LH‐RH analog	45	11.3
Aromatase inhibitor ± LH‐RH analog	211	53.0
Everolimus	4	1
Other	4	1

Abbreviations: ER, estrogen receptor; PgR, progesterone receptor; LH‐RH, luteinizing; ET, endocrine therapy; hormone‐releasing hormone; Lung^w/o liver^, lung metastasis without liver involvement; Liver^w/o lung^, liver metastasis without lung involvement.

a For premenopausal women, fulvestrant was given upon the administration of LH‐RH.

b Patients with stage IV breast cancer at initial diagnosis were excluded (n = 46).

c Patients with baseline brain metastases all received local brain radiotherapy, and there was no clinical evidence of disease progression at the time of fulvestrant administration.

d Includes patients with baseline disease site of adrenal glands (n = 4), peritoneum (n = 3), esophagus (n = 1) and pancreas (n = 1).

Thirty‐two patients (8.0%) were completely endocrine therapy naïve, while 145 patients (36.4%) had not received previous endocrine treatment for metastatic disease. Of those who had received previous endocrine treatment for metastatic disease, the majority (238 out of 253) had been exposed to an AI. The last endocrine therapy prior to fulvestrant treatment was an AI for 211 patients (53.0%) and an antiestrogen for 45 patients (11.3%). In addition, 195 (49.0%) patients had undergone chemotherapy for metastatic disease prior to treatment with fulvestrant.

### 3.2 Efficacy

**Figure 2:**
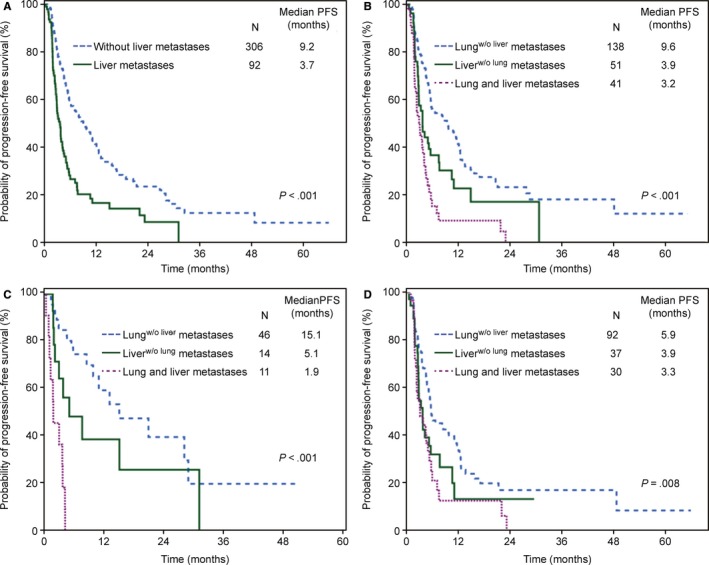
Kaplan‐Meier estimates of progression‐free survival in different subgroups. (A) Patients with or without liver metastases and (B) patients with visceral metastases divided into three subgroups based on metastatic sites. Progression‐free survival in the three subgroups after fulvestrant as first‐line therapy (C) and later‐line therapy (D). Lung^w/o liver^, lung metastasis without liver involvement; Liver^w/o lung^, liver metastasis without lung involvement; HR: hazard ratios

The median follow‐up duration was 26 months. At the time of the analysis, despite immature survival data, 253 patients (63.6%) had experienced disease progression. The overall median PFS was 6.8 months. Significantly longer PFS was observed in patients with nonvisceral disease than in patients with visceral disease (HR 1.33, 95% CI 1.03‐1.72, *P* = .029), with a median PFS of 9.2 months and 5.6 months, respectively. Meanwhile, patients with liver metastases (N = 92) experienced significantly worse PFS than those without liver involvement (N = 306) (3.7 vs. 9.2 months, *P* < .001; HR 2.17 95% CI 1.65‐2.85, *P* < .001) (Figure 2A). Furthermore, similar PFS was observed in patients with nonvisceral disease and those with lung^w/o liver^ metastases disease (9.2 vs. 9.6 months, *P* = .86).

The basic characteristics and prior treatments were balanced among the three subgroups (Table 2). Kaplan‐Meier curves revealed significantly improved PFS in patients with lung^w/o liver^ metastases compared to that in patients with liver^w/o lung^ metastases or both lung and liver metastases (liver^w/o lung^ metastases compared to lung^w/o liver^ metastases: HR 1.70, 95% CI 1.15‐2.51, *P* = .008; both lung and liver metastases compared to lung^w/o liver^ metastases: HR 2.85, 95% CI 1.93‐4.22, *P* < .001). The median PFS in patients with lung^w/o liver^ metastases, patients with liver^w/o lung^ metastases, and patients with both lung and liver metastases was 9.6, 3.9 and 3.2 months, respectively (Figure 2B). It is noteworthy that PFS was significantly longer in patients with only lung metastases than in patients with both liver and lung metastases (9.6 vs. 3.2 months, *P* < .001). However, similar PFS was observed in patients with only liver metastases and patients with both lung and liver metastases (3.9 vs. 3.2 months, *P* = .367).

**Table 2:** Baseline covariates and subgroups by different sites of visceral metastasis

Characteristics	Lung^w/o liver^ metastasis (n = 138)		Liver^w/o lung^ metastasis (n = 51)		Lung and liver metastasis (n = 41)	
	n	%	n	%	n	%
Median age, years	55 (31‐85)		57 (30‐85)		57 (31‐79)	
Menopausal status						
Premenopausal	23	16.7	10	19.6	10	24.4
Postmenopausal	115	83.3	41	80.4	31	75.6
Disease‐free interval						
≤5 years	50	36.2	26	51.0	17	41.5
>5 years	77	55.8	19	37.3	22	53.7
ER status						
Positive	137	99.3	49	96.1	41	100.0
Negative	1	0.7	2	3.9	0	0.0
PgR status				0.0		
Positive	97	70.3	38	74.5	27	65.9
Negative	34	24.6	11	21.6	13	31.7
Unknown	7	5.1	2	3.9	1	2.4
No. of disease sites						
1	25	18.1	10	19.6	0	0.0
≥2	113	81.9	41	80.4	41	100.0
Other metastatic sites						
Bone	70	50.7	31	60.7	22	53.6
Pleura	21	15.2	3	5.8	4	9.7
Brain	6	4.3	1	1.9	2	4.8
Soft tissue	69	50.0	20	39.2	24	58.5
De novo metastatic disease	10	7.2	6	11.8	2	4.9
Prior ET for metastatic disease						
No	46	33.3	14	27.5	11	26.8
Yes	92	66.7	37	72.5	30	73.2
Prior sensitivity to ET						
Primary resistance	26	18.8	19	37.3	10	24.4
Secondary resistance	102	73.9	29	56.9	29	70.7
Prior chemotherapy for metastatic disease						
No	68	49.3	14	27.5	9	22.0
Yes	70	50.7	37	72.5	32	78.0

Abbreviations: ER, estrogen receptor; PgR, progesterone receptor; ET, endocrine therapy; Lung^w/o liver^ metastasis, lung metastasis without liver involvement; Liver^w/o lung^ metastasis, liver metastasis without lung involvement.

On this basis, we further evaluated the efficacy of first‐ and later‐line treatment with fulvestrant in the three subgroups (Figure 2C,D). For patients receiving first‐line fulvestrant treatment, the HRs were as follows: liver^w/o lung^ metastases compared to lung^w/o liver^ metastases, HR 1.96, 95% CI 0.91‐4.24, *P* = .086; and both lung and liver metastases compared to lung^w/o liver^ metastases, HR 10.4, 95% CI 4.4‐24.6, *P* < .001. For patients receiving later‐line fulvestrant treatment, the HRs were as follows: liver^w/o lung^ metastases compared to lung^w/o liver^ metastases, HR 1.60, 95% CI 1.01‐2.54, *P* = .044; and both lung and liver metastases compared to lung^w/o liver^ metastases, HR 1.92, 95% CI 1.21‐3.03, *P* = .005.

### 3.3 Prognostic factors and their associations with the outcome

The results of the PFS analyses using Cox proportional hazard regression are shown in Table 3. DFI, bone‐only metastases, liver metastases, being endocrine therapy naïve, prior endocrine therapy for MBC and prior chemotherapy for MBC were significantly related to PFS (all *P* < .05). When we further corrected for these factors in the multivariate analysis, the results revealed that patients who had not received previous chemotherapy for advanced disease (HR 1.93, 95% CI 1.32‐2.82, *P* = .001), patients without liver metastases (HR 1.51, 95% CI 1.05‐2.18, *P* = .027) and patients with a DFI greater than 5 years (HR 1.42, 95% CI 1.01‐1.99, *P* = .043) experienced significantly longer PFS than their counterparts.

**Table 3:** Univariate and multivariate analysis of progression‐free survival by prespecified stratification factors

Variables	n	Univariate			Multivariate		
		Median^a^	95% CI	*P* b	HR	95% CI	*P* b
Menopausal status							
Premenopausal	84	11.0	6.4‐15.7		–	–	–
Postmenopausal	314	5.9	4.7‐7.1	.052	–	–	–
Disease‐free interval							
>5 years	181	8.6	5.8‐11.5		1		
≤5 years	171	4.8	3.6‐6.0	**.003**	1.42	1.01‐1.99	**.043**
PgR status							
Positive	285	6.9	5.3‐8.6		–	–	–
Negative + UK	113	5.5	4.6‐6.3	.107	–	–	–
Bone‐only metastasis							
Yes	61	10.9	2.7‐19.0		1		
No	337	5.8	4.8‐6.8	**.002**	1.69	0.91‐2.80	.06
Metastatic sites							
Nonvisceral	165	9.2	6.7‐11.7		1		
Lung^w/o liver^	138	9.6	5.3‐13.9	.860	–	–	–
Liver	92	3.7	2.9‐4.5	**<.001**	1.51	1.05‐2.18	**.027**
ET naïve							
Yes	32	26.8	NE‐59.3		1		
No	366	6.0	4.9‐7.2	**.01**	2.12	0.99‐4.54	.052
Prior ET for metastatic disease							
0	145	11.0	5.5‐16.6		1		
≥1	253	5.6	4.9‐6.3	**.002**	0.91	0.62‐1.34	.645
Sensitivity to prior ET							
Primary resistance	71	4.0	2.9‐5.0		–	–	–
Secondary resistance	295	7.0	5.6‐8.3	.05	–	–	–
Prior chemotherapy for metastatic disease							
0	203	9.9	6.5‐13.2		1		
≥1	195	4.7	3.9‐5.5	**<.001**	1.93	1.32‐2.82	**.001**

Abbreviations: PgR, progesterone receptor; UK, unknown; HR, hazard ratio; 95% CI, 95% confidence interval; ET, endocrine therapy; NE, not estimable; Lung^w/o liver^, lung metastasis without liver involvement.

a Median PFS in months.

b *P* < .05 was considered significant; significant values are presented in bold.

## 4 DISCUSSION

In the current study analyzing MBC patients treated with fulvestrant 500 mg, PFS was similar in patients with lung^w/o liver^ metastases and those with nonvisceral metastases and much longer than that in patients with liver^w/o lung^ metastases or both lung and liver metastases.

Our analysis included only patients treated with fulvestrant 500 mg and excluded those treated with fulvestrant 250 mg or other medications concomitantly. Previously, the phase II study FINDER2 was among the first to demonstrate the superior therapeutic efficacy of fulvestrant 500 mg compared to that of fulvestrant 250 mg,^12^ and the phase III prospective trial CONFIRM further showed that fulvestrant 500 mg was superior to fulvestrant 250 mg, as indicated by a significant increase in PFS and a corresponding clinically meaningful improvement in benefit vs. risk.^13^ Hence, only patients treated with the currently approved and now standard dose of fulvestrant 500 mg were analyzed in this study.

The randomized phase III FALCON trial proved fulvestrant 500 mg to be superior to anastrozole in treating postmenopausal hormone receptor‐positive MBC patients, yet its subgroup analysis did not reveal superior therapeutic efficacy for fulvestrant in the treatment of visceral disease.^3^ One explanation may be that the lack of a distinction among visceral sites in the visceral disease subgroup and their varying prognostic values resulted in an underestimation of the efficacy of fulvestrant. Our study demonstrates the varying prognostic values of different metastatic sites and indicates that patients with liver^w/o lung^ metastases and those with lung^w/o liver^ metastases did not benefit equally from fulvestrant treatment. Our results are in accordance with a recently published meta‐analysis by Robertson et al,^14^ which showed that patients with advanced hormone receptor + breast cancer with nonvisceral metastasis and visceral non‐liver metastasis had significantly better outcomes on endocrine therapy than patients with visceral liver metastasis.

To the best of our knowledge, this is the first study to differentiate between visceral metastatic sites and reveal a significant correlation between the visceral organs involved and the patient's prognosis after endocrine treatment. We hypothesize that there are distinct differences in the prognoses of patients with different sites of metastasis. This hypothesis is supported by a prognostic score for MBC published by Regierer et al,^11^ which identified the site of metastasis as one of the significant independent prognostic factors of overall survival. Their prognostic score assigned varying points to different metastatic sites; the lungs, bone, soft tissue and effusion were assigned 4 points each, whereas the liver was assigned 7 points, the brain 8 points and the bone marrow 10 points. We can infer from the point values that lung metastases had a relatively small adverse effect on patient prognosis. In addition, univariate analysis of pooled data from the MONARCH 2 and 3 studies did not identify lung metastasis as a prognostic factor, while liver metastasis was found to be a significant prognostic factor, and a significant difference in the median PFS was observed between patients with and without baseline liver metastasis after fulvestrant treatment.^15^ Similarly, a retrospective study from Japan regarding the efficacy of fulvestrant against MBC also found the presence of liver metastasis to be significantly correlated with poorer PFS.^16^

Aside from the metastatic site, the DFI and prior chemotherapy for metastatic disease were also significant prognostic factors in our study, which is in agreement with the results of a meta‐analysis that reported significantly better time to progression/PFS in patients with a longer time from diagnosis to recurrence after fulvestrant treatment.^17^ In clinical practice, patients with visceral metastases are more likely to receive initial chemotherapy^11^; however, ER‐positive MBC patients are more likely to respond to hormonal therapy when it is administered before chemotherapy.^18^ Our results reflect a similar conclusion, with patients who had not received prior chemotherapy for MBC exhibiting a significantly longer median PFS than those who had received prior chemotherapy.

Furthermore, univariate analysis revealed that the line of fulvestrant treatment was significantly associated with PFS; however, the same finding was not revealed by multivariate analysis. This may be due in part to our modest sample size and other confounding factors associated with retrospective analyses. The median PFS of patients treated with fulvestrant as a first‐line endocrine therapy was 11.0 months, shorter than that reported in the FIRST and FALCON studies.^3^, ^19^ One possible explanation is that the majority of patients in our study had previously received adjuvant endocrine therapy, and some had received chemotherapy for metastatic disease before starting fulvestrant.

Inevitably, our study had limitations. First, our study was performed using retrospective datasets rather than prospective cohorts, and sampling biases may have been introduced. Second, while the sample size of our study was substantial, with 58.5% of patients analyzed having visceral metastases and undergoing endocrine therapy, our results are from a single center and should be interpreted with caution.

## 5 CONCLUSIONS

The present study investigated the prognostic factors associated with improved PFS after fulvestrant 500 mg treatment in a population of Chinese breast cancer patients. Our results demonstrate that patients with liver metastases respond poorly to fulvestrant; in contrast, the efficacy of fulvestrant treatment in patients with lung^w/o liver^ metastases was comparable to that in patients with nonvisceral metastases. Therefore, we suggest that categorizing MBC patients into those with liver and non‐liver metastases may better reflect patient prognosis than the current categorization of patients with visceral and nonvisceral metastases. However, further studies are necessary to determine whether patients with hormone receptor‐positive/HER2‐negative MBC would benefit from being treated differentially according to the site of visceral metastasis, and we await the results of subgroup analyses from prospective, multicenter trials for more supporting evidence.

## CONFLICTS OF INTEREST

The authors have declared no conflicts of interest.

## References

[1] Lobbezoo D , van Kampen R , Voogd AC , et al. Prognosis of metastatic breast cancer subtypes: the hormone receptor/HER2‐positive subtype is associated with the most favorable outcome. Breast Cancer Res Treat. 2013;141(3):507‐514. 24104881 10.1007/s10549-013-2711-y

[2] Cardoso F , Costa A , Senkus E , et al. 3rd ESO‐ESMO International Consensus Guidelines for Advanced Breast Cancer (ABC 3). Ann Oncol. 2017;28(12):3111. 28327998 10.1093/annonc/mdx036 PMC5834023

[3] Robertson J , Bondarenko IM , Trishkina E , et al. Fulvestrant 500 mg versus anastrozole 1 mg for hormone receptor‐positive advanced breast cancer (FALCON): an international, randomised, double‐blind, phase 3 trial. Lancet. 2016;388(10063):2997‐3005. 27908454 10.1016/S0140-6736(16)32389-3

[4] Finn RS , Martin M , Rugo HS , et al. Palbociclib and letrozole in advanced breast cancer. N Engl J Med. 2016;375(20):1925‐1936. 27959613 10.1056/NEJMoa1607303

[5] Hortobagyi GN , Stemmer SM , Burris HA , et al. Ribociclib as first‐line therapy for HR‐positive, advanced breast cancer. N Engl J Med. 2016;375(18):1738‐1748. 27717303 10.1056/NEJMoa1609709

[6] Goetz MP , Toi M , Campone M , et al. MONARCH 3: abemaciclib as initial therapy for advanced breast cancer. J Clin Oncol. 2017;35(32):3638‐3646. 28968163 10.1200/JCO.2017.75.6155

[7] Robertson J , Lindemann J , Garnett S , et al. A good drug made better: the fulvestrant dose‐response story. Clin Breast Cancer. 2014;14(6):381‐389. 25457991 10.1016/j.clbc.2014.06.005

[8] Di Leo A , Jerusalem G , Petruzelka L , et al. Results of the CONFIRM phase III trial comparing fulvestrant 250 mg with fulvestrant 500 mg in postmenopausal women with estrogen receptor‐positive advanced breast cancer. J Clin Oncol. 2010;28(30):4594‐4600. 20855825 10.1200/JCO.2010.28.8415

[9] Zhang Q , Shao Z , Shen K , et al. Fulvestrant 500 mg vs 250 mg in postmenopausal women with estrogen receptor‐positive advanced breast cancer: a randomized, double‐blind registrational trial in China. Oncotarget. 2016;7(35):57301‐57309. 27359058 10.18632/oncotarget.10254 PMC5302990

[10] Ellis MJ , Llombart‐Cussac A , Feltl D , et al. Fulvestrant 500 mg versus anastrozole 1 mg for the first‐line treatment of advanced breast cancer: overall survival analysis from the phase II FIRST study. J Clin Oncol. 2015;33(32):3781‐3787. 26371134 10.1200/JCO.2015.61.5831 PMC4737861

[11] Regierer AC , Wolters R , Ufen MP , et al. An internally and externally validated prognostic score for metastatic breast cancer: analysis of 2269 patients. Ann Oncol. 2014;25(3):633‐638. 24368402 10.1093/annonc/mdt539 PMC4433507

[12] Pritchard KI , Rolski J , Papai Z , et al. Results of a phase II study comparing three dosing regimens of fulvestrant in postmenopausal women with advanced breast cancer (FINDER2). Breast Cancer Res Treat. 2010;123(2):453‐461. 20632084 10.1007/s10549-010-1022-9

[13] Di Leo A , Jerusalem G , Petruzelka L , et al. Final overall survival: fulvestrant 500 mg vs 250 mg in the randomized CONFIRM trial. J Natl Cancer Inst. 2014;106(1):djt337. 24317176 10.1093/jnci/djt337 PMC3906991

[14] Robertson J , Lichfield J , Bradbury I , Campbell C . Meta‐analyses of visceral versus non‐visceral metastases treated by selective estrogen receptor modulator, aromatase inhibitor, and selective estrogen receptor degrader agents as first‐line endocrine therapy for hormone receptor‐positive breast cancer. SABCS 2018. 2018:P4‐13‐1.

[15] Goetz MP , O'Shaughnessy J , Sledge GW Jr , et al. The benefit of abemaciclib in progostic subgroups: an exploratory analysis of combined data from the MONARCH 2 and 3 studies. San Antonio Breast Cancer Symposium. 2017;Abstract;GS06_2.

[16] Araki K , Ishida N , Horii R , et al. Efficacy of fulvestrant 500 mg in Japanese postmenopausal advanced/recurrent breast cancer patients and factors associated with prolonged time‐to‐treatment failure. Expert Opin Pharmacother. 2015;16(17):2561‐2568. 26558799 10.1517/14656566.2015.1107042

[17] Graham J , Pitz M , Gordon V , Grenier D , Amir E , Niraula S . Clinical predictors of benefit from fulvestrant in advanced breast cancer: a meta‐analysis of randomized controlled trials. Cancer Treat Rev. 2016;45:1‐6. 26922660 10.1016/j.ctrv.2016.02.004

[18] Freedman O , Amir E , Dranitsaris G , et al. Predicting benefit from fulvestrant in pretreated metastatic breast cancer patients. Breast Cancer Res Treat. 2009;118(2):377‐383. 19551499 10.1007/s10549-009-0452-8

[19] Robertson JF , Lindemann JP , Llombart‐Cussac A , et al. Fulvestrant 500 mg versus anastrozole 1 mg for the first‐line treatment of advanced breast cancer: follow‐up analysis from the randomized 'FIRST' study. Breast Cancer Res Treat. 2012;136(2):503‐511. 23065000 10.1007/s10549-012-2192-4

